# Activation of PPAR*γ*/P53 signaling is required for curcumin to induce hepatic stellate cell senescence

**DOI:** 10.1038/cddis.2016.92

**Published:** 2016-04-14

**Authors:** H Jin, N Lian, F Zhang, L Chen, Q Chen, C Lu, M Bian, J Shao, L Wu, S Zheng

**Affiliations:** 1 Department of Pharmacology, School of Pharmacy, Nanjing University of Chinese Medicine, Nanjing 210023, China; 2 Jiangsu Key Laboratory for Pharmacology and Safety Evaluation of Chinese Materia Medica, Nanjing University of Chinese Medicine, Nanjing 210023, China; 3 Department of Pharmacy, School of Pharmacy, Nanjing University of Chinese Medicine, Nanjing 210023, China

## Abstract

Activation of quiescent hepatic stellate cells (HSCs) is the major event in hepatic fibrogenesis, along with enhancement of cell proliferation and overproduction of extracellular matrix. Although inhibition of cell proliferation and induction of apoptosis are potential strategies to block the activation of HSCs, a better understanding of the senescence of activated HSCs can provide a new therapeutic strategy for prevention and treatment of liver fibrosis. The antioxidant curcumin, a phytochemical from turmeric, has been shown to suppress HSC activation *in vitro* and *in vivo*. The current work was aimed to evaluate the effect of curcumin on senescence of activated HSCs and to elucidate the underlying mechanisms. In this study, curcumin promoted the expression of senescence marker Hmga1 in rat fibrotic liver. In addition, curcumin increased the number of senescence-associated *β*-galactosidase-positive HSCs *in vitro*. At the same time, curcumin induced HSC senescence by elevating the expression of senescence markers P16, P21 and Hmga1, concomitant with reduced abundance of HSC activation markers *α*-smooth muscle actin and *α*1(I)-procollagen in cultured HSCs. Moreover, curcumin affected the cell cycle and telomerase activity. We further demonstrated that P53 pharmacological inhibitor pifithrin-*α* (PFT-*α*) or transfection with P53 siRNA abrogated the curcumin-induced HSC senescence *in vitro*. Meanwhile, curcumin disruption of P53 leading to increased senescence of activated HSCs was further verified *in vivo*. Further studies indicated that curcumin promoted the expression of P53 through a PPAR*γ* activation-dependent mechanism. Moreover, promoting PPAR*γ* transactivating activity by a PPAR*γ* agonist 15d-PGJ2 markedly enhanced curcumin induction of senescence of activated HSCs. However, the PPAR*γ* antagonist PD68235 eliminated curcumin induction of HSC senescence. Taken together, our results provided a novel insight into the mechanisms underlying curcumin inhibition of HSC activation through inducing senescence.

Hepatic stellate cells (HSCs), previously known as vitamin A-storing cells or Ito cells, are the major effector cells in the development of liver fibrosis.^1, 2^ Upon the liver injury, quiescent HSCs become activated and trans-differentiate into myofibroblast-like cells, which are characterized by enhanced cell growth and undergo profound phenotypic changes, including *de novo* expression of *α*-smooth muscle actin (*α*-SMA) and production of large amounts of collagens.^3, 4^ It has been recognized that suppression of the activation of HSCs can prevent and treat hepatic fibrosis. Typically, inhibition of cell proliferation and induction of apoptosis are potential strategies to block the activation of HSCs, but induction of activated HSC senescence could be a new therapeutic strategy for prevention and treatment of liver fibrosis. Recent evidence has revealed that the senescence of activated HSCs was an important step in limiting the fibrogenic response to liver tissue damage.^5, 6^

Cellular senescence is described as an antiproliferative program that leads to permanent growth arrest in the cell.^7^ Cellular senescence is typically characterized by telomere shortening or telomere dysfunction,^8, 9^ activation of genes at the INK4a/ARF locus^10^ and accumulation of DNA damage.^7, 11^ Senescent cells undergo a stable growth arrest mediated by interplay of multiple pathways. Recently, accumulating evidence has shown that the tumor suppressor P53 can regulate cellular senescence.^12^ The P53 can lead to cell cycle arrest, DNA repair and apoptosis predominantly when it becomes transcriptionally active in response to DNA damage, oncogene activation and hypoxia.^13^ Deletion of P53 is found to reduce HSC senescence leading to extensive liver fibrosis.^5^ Moreover, peroxisome proliferator activated receptor *γ* (PPAR*γ*), one of the PPAR isoforms, is highly expressed in quiescent HSCs in the normal liver, but its expression and activity are dramatically diminished during HSC activation *in vitro* and *in vivo*.^14, 15^ A recent study has found that PPAR*γ* could play a potential role in liver fibrosis, which could regulate HSC senescence.^16^

The polyphenolic antioxidant curcumin, a primary active component of the rhizome of the plant turmeric (*Curcuma longa* Linn), possesses antiproliferative, antioxidant, anti-inflammatory, antiangiogenic and antitumor effects. Previous reports demonstrated that curcumin inhibited activation of HSC *in vitro* by suppressing cell growth and inhibiting production of extracellular matrix (ECM) components.^17^ Curcumin promoted the expression of PPAR*γ* and stimulated the activation of PPAR*γ*, which was a precondition for curcumin to inhibit HSC activation.^18^ The purpose of the present study was to investigate the signal transduction pathways involved in curcumin induction of activated HSC senescence. We hypothesized that modulation of PPAR*γ* could contribute to curcumin induction of HSC senescence through promoting the expression of P53. We therefore performed *in vivo* and *in vitro* experiments to test the hypothesis.

## Results

### Curcumin promoted HSC senescence and P53 expression in rat fibrotic liver

**Figure 1:**
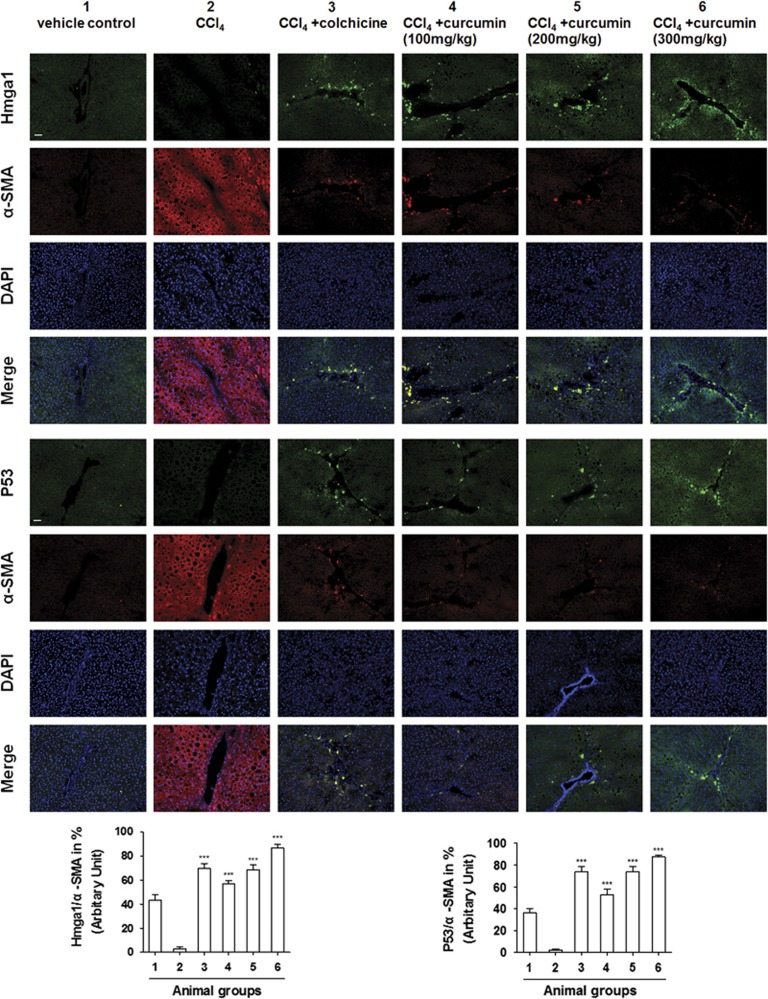
Curcumin promoted HSC senescence and P53 expression in rat fibrotic liver. Rats were grouped as follows: group 1, vehicle control (no CCl_4_, no treatment); group 2, model group (with CCl_4_, no treatment); group 3, colchicine-treated group (0.1 mg/kg+CCl_4_); group 4, curcumin-treated group (100 mg/kg+CCl_4_); group 5, curcumin-treated group (200 mg/kg+CCl_4_); and group 6, curcumin-treated group (300 mg/kg+CCl_4_). Liver sections were stained with immunofluorescence using antibodies against Hmga1 and P53. Antibody against *α*-SMA was used to specifically stain HSCs, and DAPI to stain the nucleus. Bar graphs showed the percentage quantitation of fluorescent images of Hmga1 or P53-positive cells in total activated HSCs (*α*-SMA as a marker) using the software Image J as shown in the figure. ***P<0.001 *versus* group 2. Scale bar, 50 *μ*m

Our previous data have sufficiently demonstrated that curcumin protected the liver from histological injury, pathological angiogenesis and fibrogenesis induced by chronic CCl_4_ injection in rats.^19, 20, 21^ In the present study, we examined the senescence marker in rat fibrotic liver firstly. Results from immunofluorescence staining showed that curcumin increased the expression of senescence marker Hmga1^22^ in HSCs concomitant with the expression of *α*-SMA, the marker of HSC activation (Figure 1). To test the role of P53 in curcumin induction of HSC senescence *in vivo*, we examined the levels of P53 in rat fibrotic liver. Chronic CCl_4_ intoxication reduced the production of P53 in rat fibrotic liver, but curcumin significantly increased the expression of P53 in liver tissues (Figure 1).

### Curcumin promoted activated HSC senescence *in vitro*

**Figure 2:**
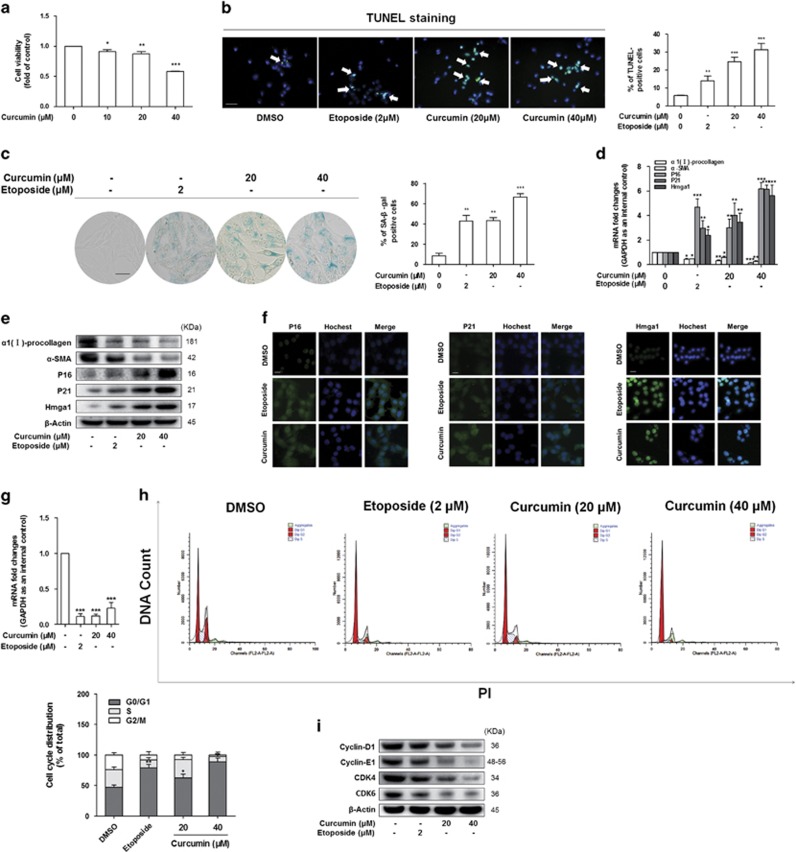
Curcumin promoted senescence of activated HSC *in vitro*. HSCs were treated with DMSO (0.02%, w/v), etoposide or curcumin at indicated concentrations for 24 h. (**a**) Cell Count Kit-8 analysis of the ability of cell proliferation. *P<0.05 *versus* DMSO, **P<0.01 *versus* DMSO, ***P<0.001 *versus* DMSO. (**b**) TUNEL staining for evaluating apoptosis. Green fluorescence indicates apoptotic cells. Percentages of TUNEL-positive cells were determined. Data are represented as mean±S.D. **P<0.01 *versus* DMSO, ***P<0.001 *versus* DMSO. Scale bar, 50 *μ*m. (**c**) Senescence *β*-galactosidase staining analysis. Percentages of cells positive for SA-*β*-Gal were scored; the averages±S.D. of data from at least three separate experiments are shown. **P<0.01 *versus* DMSO, ***P<0.001 *versus* DMSO. Scale bar, 200 *μ*m. (**d**) Real-time PCR analyses of genes relevant to fibrogenesis and senescence, including *α*-SMA, *α*1(I)-procollagen and P16, P21 and Hmga1, respectively. Data are represented as mean±S.D. Significance: *P<0.05 *versus* DMSO, **P<0.01 *versus* DMSO, ***P<0.001 *versus* DMSO. (**e**) Western blot analyses of protein expression of fibrogenic molecules *α*-SMA, *α*1(I)-procollagen and senescence molecules P16, P21 and Hmga1. (**f**) Immunofluorescence using antibody against P16, P21, Hmga1. Hoechst reagent was used to stain the nucleus. Scale bar, 10 *μ*m. (**g**) Real-time PCR analysis of the mRNA levels of telomerase (TERT). Data are represented as mean±S.D. ***P<0.001 *versus* DMSO. (**h**) Cell cycle analysis by flow cytometry. Percentages of cell cycle distributions were determined. Data are represented as mean±S.D. *P<0.05 *versus* DMSO, **P<0.01 *versus* DMSO. (**i**) Western blot analyses of cell cycle-regulatory proteins cyclin D1, cyclin E1, CDK4 and CDK6

We used cultured HSCs to test whether curcumin had direct effects on HSC activation and senescence. Etoposide is a recognized DNA-damaging agent, which can promote cellular senescence, and thus here Etoposide treatment served as a positive control. We investigated the effects of curcumin on cell proliferation and apoptosis in cultured HSCs initially. Cell Counting Kit-8 analysis indicated that the ability of cell proliferation was inhibited by treatment with curcumin (Figure 2a). TUNEL staining revealed that curcumin had effects on HSC apoptosis (Figure 2b). Next, we found that curcumin treatment increased the number of senescence-associated *β*-galactosidase (SA-*β*-Gal^+^)-positive HSCs (Figure 2c). Meanwhile, curcumin exposure decreased the mRNA and protein levels of *α*-SMA and *α*1(I)-procollagen in cultured HSCs (Figures 2d and e), suggesting that curcumin inhibited HSC activation. Our further results showed that curcumin promoted activated HSC senescence. Real-time PCR, western blot and immunofluorescence analyses of senescence-associated genes consistently showed that curcumin treatment upregulated the expression of senescence markers P16, P21^23^ and Hmga1 (Figures 2d–f). Additional experiments were performed to verify the role of telomerase activity in curcumin induction of activated HSC senescence. We found that the telomerase activity was decreased in activated HSCs by curcumin (Figure 2g).

A well-known feature of cellular senescence is cell cycle arrest, which largely accounts for the growth inhibition in senescent cells.^24^ Next, we examined the cell cycle distribution by a flow cytometer. As shown in Figure 2h, HSCs treated with curcumin showed significantly higher proportions of G1 cells and lower proportions of S cells compared with untreated HSCs. Cell cycle is influenced by multiple cyclins and cyclin-dependent kinases (CDKs). The cyclin D1/CDK4 complex together with the cyclin E1/CDK6 complex promote the G0- to S-phase transition. Western blot analyses indicated that curcumin downregulated the four molecules (Figure 2i). These data revealed that curcumin arrested HSCs at the G0/G1 checkpoint by inducing HSC senescence in activated HSCs.

### Curcumin induced activated HSC senescence via a P53-dependent mechanism

**Figure 3:**
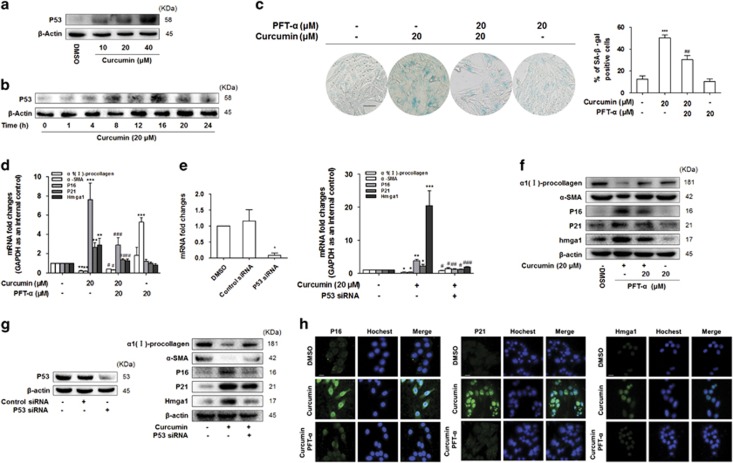
Curcumin induced senescence of activated HSC via a P53-dependent mechanism. (**a** and **b**) Western blot analyses of protein expression of P53. HSCs were treated with DMSO (0.02%, w/v) or curcumin at the indicated concentrations for 24 h or at 20 *μ*M for the indicated time periods. (**c**) Senscence *β*-galactosidase staining analysis. HSCs were treated with DMSO (0.02%, w/v), curcumin (20 *μ*M) and/or PFT-*α* (20 *μ*M) for 24 h. PFT-*α*: a specific P53 inhibitor. Percentages of cells positive for SA-*β*-Gal were scored; the averages±S.D. of data from at least three separate experiments are shown. ***P<0.001 *versus* DMSO, ^##^*P*<0.01 *versus* curcumin. Scale bar, 200 *μ*m (**d** and **e**) Real-time PCR analyses of genes relevant to fibrogenesis and senescence, including *α*-SMA, *α*1(I)-procollagen and P16, P21, Hmga1, respectively. Data are represented as mean±S.D. Significance: *P<0.05 *versus* DMSO or control siRNA, **P<0.01 *versus* DMSO or control siRNA, ***P<0.001 *versus* DMSO or control siRNA, ^#^*P*<0.05 *versus* curcumin, ^##^*P*<0.01 *versus* curcumin, ^###^*P*<0.001 *versus* curcumin. (**f** and **g**) Western blot analyses of protein expression of fibrogenic molecules *α*-SMA, *α*1(I)-procollagen and senescence molecules P16, P21, Hmga1. (**h**) Immunofluorescence using antibody against P16, P21 and Hmga1. Hoechst reagent was used to stain the nucleus. Scale bar, 10 *μ*m

P53 is the major mediator of cell cycle arrest and senescence in response to a series of cellular damage.^25^ As illustrated in Figures 3a and b, the expression of P53 was dose- and time-dependently increased by curcumin. On the other hand, P53 inhibitor PFT-*α* decreased the number of SA-*β*-Gal^+^ HSCs, compared with the cells treated with curcumin alone (Figure 3c). At the same time, analyses of real-time PCR, western blot and immunofluorescence staining displayed that the P53 inhibitor PFT-*α* enhanced the pro-fibrotic effects and weakened the pro-senescence effects of curcumin (Figures 3d, f and h). In addition, we tested the role of P53 with a different strategy that downregulated the P53 levels by siRNA silencing, and the results with P53 siRNA transfection were consistent with the experiments from treatment with PFT-*α* (Figures 3e and g). Taken together, these findings consistently revealed that curcumin promoted the senescence of activated HSCs by inducing the expression of P53.

### P53 mediated curcumin-induced activated HSC senescence *in vivo*

**Figure 4:**
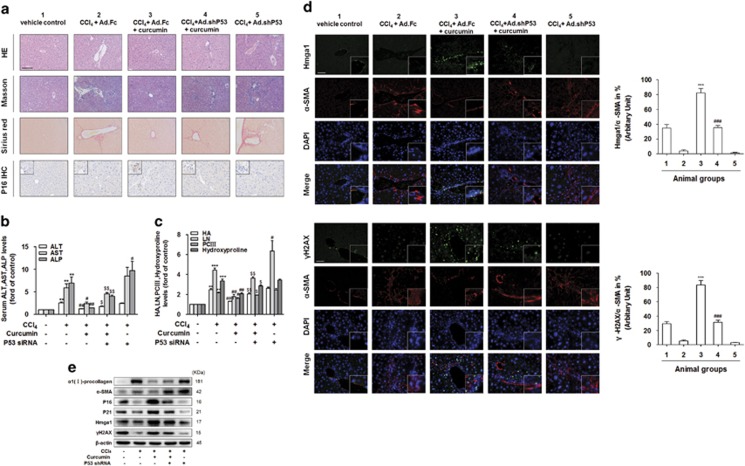
P53 mediated curcumin-induced senescence of activated HSC *in vivo*. Mice were randomly separated into five groups: group 1, vehicle control (no CCl_4_, no treatment); group 2 (with CCl_4_+Ad.Fc); group 3 (with CCl_4_+curcumin+Ad.Fc); group 4 (with CCl_4_+curcumin+Ad.shP53); and group 5 (with CCl_4_+Ad.shP53). Mice were injected with CCl_4_ (0.5 ml per 100 g body weight, i.p., twice a week) or curcumin (300 mg/kg body weight, once per 2 days) for 8 weeks. Ad.shP53 or Ad.Fc (2.5 × 10^7^ pfu/g body weight, once every 2 weeks) was injected into the tail vein. Ad.Fc was used as a control virus. (**a**) Liver sections were stained with hematoxylin and eosin, Masson reagents, sirius red and P16. Representative photographs are shown. Scale bar, 100 *μ*m. (**b** and **c**) Determination of serum alanine aminotransferase (ALT), aspartate aminotransferase (AST), alkaline phosphatase (ALP), hyaluronic acid (HA), laminin (LN), procollagen type III (PCIII) and hydroxyproline levels. Data are represented as mean±S.D. Significance: **P<0.01 *versus* group 1, ***P<0.001 *versus* group 1, ^#^*P*<0.05 *versus* group 2, ^##^*P*<0.01 *versus* group 2, ^###^*P*<0.001 *versus* group 2, ^$^*P*<0.05 *versus* group 3, ^$$^*P*<0.01 *versus* group 3. (**d**) Liver sections were stained with immunofluorescence by using antibodies against Hmga1 and *γ*H2AX (a marker of DNA damage). Antibody against *α*-SMA was used to specifically stain HSCs, and DAPI to stain the nucleus. Bar graphs showed the percentage quantitation of fluorescent images of Hmga1 or *γ*H2AX-positive cells in total activated HSCs (*α*-SMA as a marker) using the software Image J as shown in (**d**). ***P<0.001 *versus* group 2, ^###^*P*<0.001 *versus* group 3. Scale bar, 50 *μ*m. (**e**) Western blot analyses of a-SMA, *α*1(I)-procollagen, P16, P21, Hmga1 and *γ*H2AX protein levels in isolated HSCs

Next, we examined whether disruption of P53 could affect the senescence of activated HSCs *in vivo*. Mice were divided into five groups and four of these groups were administrated with Ad.Fc, curcumin plus Ad.Fc, curcumin plus Ad.shP53 or Ad.shP53, respectively, throughout the 8-week period of CCl_4_ treatment. Liver fibrosis was demonstrated by histological analyses. Hematoxylin and eosin (H&E), Masson and picro-Sirius red staining showed that the inhibitory effect of curcumin on liver fibrogenesis was remarkably decreased by interrupting P53 in the model (Figure 4a). Moreover, livers derived from mice treated with curcumin plus Ad.shP53 exhibited downregulated levels of the senescence marker P16, compared with the mice treated with curcumin alone (Figure 4a). Low levels of serum alanine aminotransferase, aspartate aminotransferase and alkaline phosphatase in curcumin-treated mice were also elevated by suppression of P53 (Figure 4b). At the same time, knockdown of P53 significantly upregulated the levels of serum hyaluronic acid, laminin, procollagen type III and hydroxyproline (Figure 4c). To further confirm the above results, primary HSCs were isolated and directly used for western blot analysis of activated HSC markers *α*1(I)-procollagen and *α*-SMA. The results demonstrated that interference of P53 significantly elevated the expression of *α*1(I)-procollagen and *α*-SMA, suggesting that the effect of curcumin was at least partially reversed (Figure 4e). We then determined whether the P53 pathway contributed to curcumin promotion of P16, P21 and Hmga1 expression in HSCs *in vivo*. Liver tissues were used for fluorescent staining of Hmga1 and *γ*H2AX (a marker of DNA damage) with *α*-SMA, respectively, by double fluorescent staining (Figure 4d). Furthermore, HSCs were isolated from the liver and directly used for western blot analyses of P16, P21, Hmga1 and *γ*H2AX (Figure 4e). The results indicated that curcumin markedly increased the number of senescence marker-positive HSCs, which was partially reversed by Ad.shP53. Similar results were obtained by western blot analyses of P16, P21 and Hmga1 in primary HSCs. These data collectively suggested that the P53 pathway was involved in curcumin induction of P53 expression in HSCs *in vivo*.

### Curcumin activation of PPAR*γ* promoted transactivation of P53

**Figure 5:**
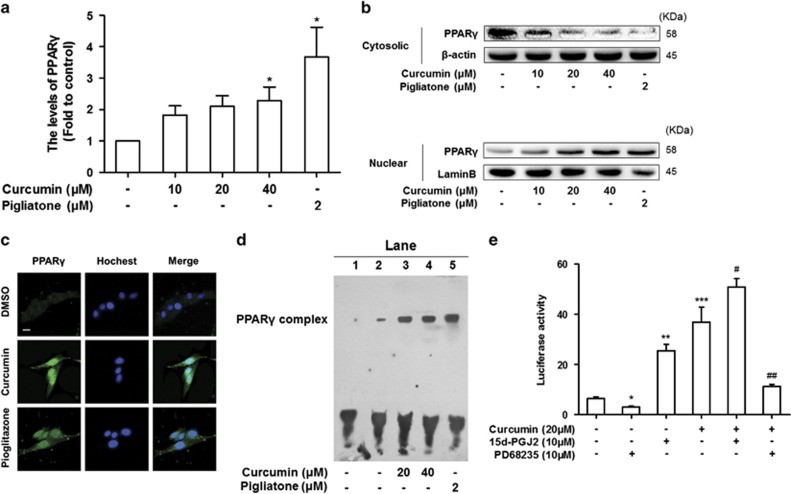
Curcumin activation of PPAR*γ* promoted transactivation of P53. HSCs were treated with DMSO (0.02%, w/v), curcumin and pigliatone at indicated concentrations for 24 h. (**a**) The levels of PPAR*γ* in culture HSC were examined by ELISA. Data are represented as mean±S.D. Significance: *P<0.05 *versus* DMSO. (**b**) Western blot analyses of protein abundance of PPAR*γ* in the cytoplasm and nucleus, respectively. (**c**) Immunofluorescence using antibody against PPAR*γ*. Hoechst reagent was used to stain the nucleus. Scale bar, 10 *μ*m. (**d**) EMSA for examining the binding capacity of PPAR*γ* to DNA sequences. Lane 1 indicates samples treated with probe alone. Lane 2 was samples treated with DMSO without curcumin, Lane 3 was samples treated with curcumin (20 *μ*M), Lane 4 was samples treated with curcumin (40 *μ*M), Lane 5 was samples treated with Pigliatone (2 *μ*M). (**e**) Passaged HSC were transfected with the pP53-Luc. Cells were then pretreated with or without 15d-PGJ2 (10 *μ*M), PD68235 (10 *μ*M), for 1 h prior to addition of curcumin (20 *μ*M) for an additional 24 h. Luciferase activities are expressed as relative units after Renilla luciferase reporter normalization (*n*=6). Data are represented as mean±S.D. Significance: *P<0.05 *versus* DMSO, **P<0.01 *versus* DMSO. ***P<0.001 *versus* DMSO, ^#^*P*<0.05 *versus* curcumin, ^##^*P*<0.01 *versus* curcumin

Previous reports indicated that curcumin inhibited HSC proliferation by inducing gene expression of PPAR*γ* in activated HSCs *in vitro*.^15^ Our current study validated this observation and showed that curcumin elevated the levels of PPAR*γ* (Figure 5a). We here assumed that activation of PPAR*γ* by curcumin might stimulate the gene expression of P53, which in turn stimulated the senescence of activated HSCs and ultimately led to reduction of live fibrosis. To test this assumption, determination of PPAR*γ* distribution by western blot analyses revealed that curcumin dose-dependently increased PPAR*γ* abundance in nucleus and decreased its abundance in the cytoplasm (Figure 5b). Immunofluorescence double staining showed that PPAR*γ* accumulated in nucleus upon curcumin treatment (Figure 5c). The interaction of PPAR*γ* with DNA sequence was also significantly increased by curcumin as demonstrated by the EMSA data (Figure 5d). To test whether PPAR*γ* interacted with P53 to regulate the senescence of activated HSCs, cells were transiently transfected with the P53-inducible luciferase reporter plasmid pP53-TA-luc, and then were treated with the PPAR*γ* antagonist PD68235 (10 *μ*M) or PPAR*γ* agonist 15d-PGJ2 (10 *μ*M) prior to the addition of curcumin. Luciferase assays demonstrated that forced expression of PPAR*γ* mimicked the curcumin induction of luciferase activity in a dose-dependent manner (Figure 5e). These results indicated that curcumin activation of PPAR*γ* induced the gene expression of P53 in activated HSCs *in vitro*.

### Activation of PPAR*γ* is required for curcumin induction of P53-dependent senescence of activated HSCs

**Figure 6:**
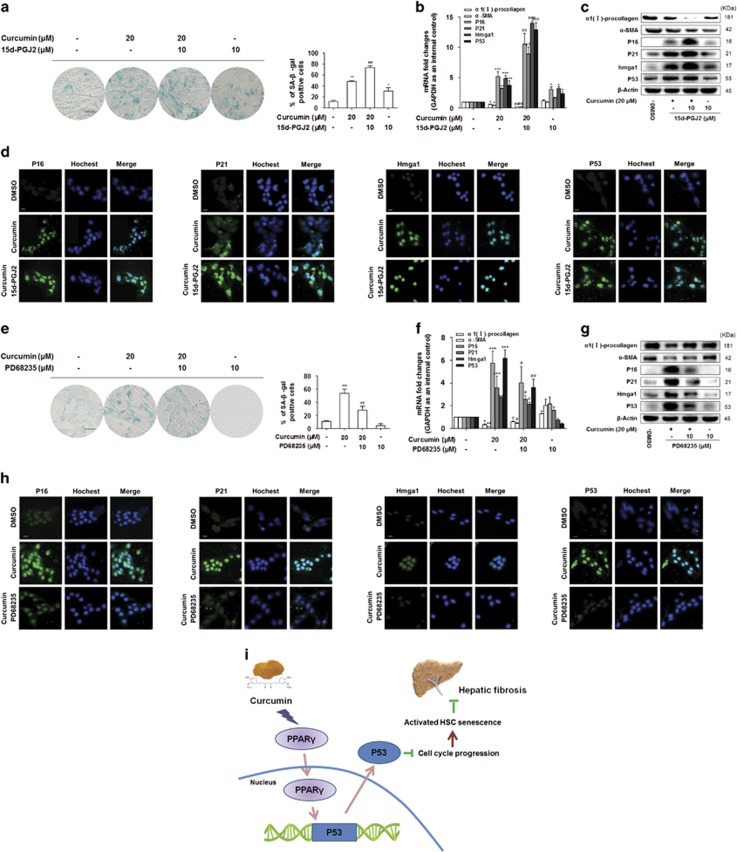
Activation of PPAR*γ* is required for curcumin induction of P53-dependent HSC senescence. HSCs were treated for 24 h with curcumin (20 *μ*M) with or without the pre-exposure to 15d-PGJ2 (10 *μ*M) and PD68235 (10 *μ*M) for 1 h. (**a** and **e**) Senescence *β*-galactosidase staining analysis. Percentages of cells positive for SA-*β*-Gal were scored; the averages±S.D. of data from at least three separate experiments are shown. *P<0.05 *versus* DMSO, **P<0.01 *versus* DMSO, ***P<0.001 *versus* DMSO, ^##^*P*<0.01 *versus* curcumin. Scale bar, 200 *μ*m. (**b** and **f**) Real-time PCR analyses of genes relevant to fibrogenesis and senescence, including *α*-SMA, *α*1(I)-procollagen and P16, P21, Hmga1 respectively. Data are represented as mean±S.D. Significance: *P<0.05 *versus* DMSO, **P<0.01 *versus* DMSO, ***P<0.001 *versus* DMSO, ^#^*P*<0.05 *versus* curcumin, ^##^*P*<0.01 *versus* curcumin, ^###^*P*<0.001 *versus* curcumin. (**c** and **g**) Western blot analyses of protein expression of fibrogenic molecules *α*-SMA, *α*1(I)-procollagen and senescence molecules P16, P21 and Hmga1. (**d** and **h**) Immunofluorescence using antibody against P16, P21 and Hmga1. Hoechst reagent was used to stain the nucleus. Scale bar, 10 *μ*m. (**b**–**d**, **f**–**h**) Real-time PCR, western blot and immunofluorescence analyses of the mRNA and protein levels of P53. Data are represented as mean±S.D. Scale bar, 10 *μ*m. (**i**) PPAR*γ* signaling played an important role in activated HSC senescence through transcriptionally upregulating P53 expression

To further test the role of PPAR*γ* in curcumin induction of HSC senescence, cultured HSCs were pretreated with 15d-PGJ2 (10 *μ*M) or PD68235 (10 *μ*M) for 1 h prior to addition of curcumin (20 *μ*M) for an additional 24 h. Treatment with 15d-PGJ2 induced a high accumulation of SA-*β*-gal^+^ HSCs, compared with the cells treated with curcumin alone (Figure 6a). We found that curcumin downregulation of *α*-SMA and *α*1(I)-procollagen, and upregulation of P16, P21 and Hmga1 were all strengthened by 15d-PGJ2 (Figures 6b and c). As expected, immunofluorescence double staining showed that the senescence markers P16, P21 and Hmga1 were highly expressed in HSCs upon 15d-PGJ2 treatment (Figure 6d). Furthermore, pretreatment of cells with PD68235 weakened the pro-senescence effects of curcumin (Figure 6e), but PD68235 abolished curcumin inhibition of *α*-SMA and *α*1(I)-procollagen and abrogated curcumin induction of P16, P21 and Hmga1 expression at both mRNA and protein levels (Figures 6f and g). Immunofluorescence double staining detecting the expression of senescence markers of P16, P21 and Hmga1 provided consistent results (Figure 6h). Moreover, we also examined the levels of P53 in the presence of PD68235 or 15d-PGJ2. The results showed that curcumin induction of P53 expression was elevated by 15d-PGJ2 treatment (Figures 6b–d). In contrast, PD68235 inhibited the stimulatory effect of curcumin on P53 expression (Figures 6f–h). These discoveries collectively suggested that PPAR*γ* played a key role in curcumin induction of HSC senescence.

## Discussion

The phenotype of senescent cells is characterized by a terminal cell cycle arrest, expression of SA-*β*-gal (a lysosomal enzyme) and induction of p16 and p21. The process of senescence was first described as a state of terminal proliferative exhaustion^26^ and was mediated by progressive telomere shortening and activation of a DNA damage response. Prior studies have discovered the existence of senescent activated HSCs during the development of fibrosis. It is considered that the senescence of activated HSCs is a spontaneous phenomenon and limits the fibrogenic response to acute tissue damage. Senescent activated HSCs reduced the secretion of ECM components, increased the secretion of ECM-degrading enzymes and enhanced immune surveillance.^6, 27^ Therefore, the senescence of activated HSCs induced by drugs may be an effective method for hepatic fibrosis treatment.

In previous studies, we have studied the mechanism of curcumin underlying its antifibrotic efficacy in depth. Growing evidence has shown that curcumin induces HSC apoptosis and our current results proved this point. The aim of the present study was to confirm whether curcumin had a regulatory effect on HSC senescence. Our results suggested that curcumin could inhibit cell proliferation and regulate the senescence of activated HSCs. On the one hand, our present animal experiments demonstrated that the senescence marker Hmga1 was co-localized with the marker of activated HSCs in rat fibrotic liver and that the number of activated HSCs was directly opposite to the level of Hmga1. On the other hand, SA-*β*-gal staining provided the evidence that senescent HSCs could be detected in curcumin-treated HSCs *in vitro*. Although P16 and P21 serve as the regulators of cell cycle, increasing evidence has demonstrated that P16 and P21 are upregulated in senescent cells.^28, 29^ Herein, we used them as the markers of cellular senescence. In this report, curcumin increased the gene expression of senescence markers P16, P21 and Hmga1 in cultured HSCs. However, the mRNA and protein levels of *α*-SMA and *α*1(I)-procollagen were significantly decreased by the curcumin treatment. These discoveries supported the possibility that induction of HSC senescence could treat liver fibrosis.

Cell cycle arrest and telomerase system dysfunction are the critical features of cellular senescence.^30^ We herein found that curcumin blocked cell cycle arrest at the G0/G1 checkpoint. Cell cycle progression is the result of the interaction between cyclins and their inhibitors CDKs.^31^ Consistently, our current data indicated that curcumin reduced the protein abundance of the G0/G1 phase-related cyclins/CDKs. At the same time, real-time PCR analyses showed that telomerase activity was decreased by curcumin treatment. It has been well-known that cellular senescence is accompanied with the reduction in telomerase activity and telomere length.^32^ Herein, our results strengthened the observation of induction of HSC senescence by curcumin, which could be a strategy for cell-fate regulation in HSCs.

Further studies revealed that the tumor suppressor P53 played an important role in induction of cellular senescence by curcumin. This raised a question of how to control HSC senescence by curcumin. In the present study, we found that curcumin dose- and time-dependently promoted the expression of P53, but the P53 inhibitor pifithrin-*α* (PFT-*α*) or P53 siRNA weakened the pro-senescence effects of curcumin in cultured HSCs. Besides, to provide further evidence for a functional role of P53 activation in the senescence of activated HSCs *in vivo*, mice were subjected to administration of Ad.Fc, curcumin plus Ad.Fc, curcumin plus Ad.shP53 or Ad.shP53. We found that curcumin's promotion effect of HSC senescence could be reversed by Ad.shP53. This result was consistent with other prior observations. In many cell types, P53 has been found to regulate the cell cycle arrest and cellular senescence.^25, 33^ Therefore, the induction of HSC senescence by P53 could be a therapeutic target for reduction of HSC activation and inhibition of profibrogenic behaviors of activated HSCs.

Cellular senescence mediated by P53 is influenced by multiple signaling pathways, such as IL-22/STAT3 pathway.^34^ There is another question of how to regulate the expression of P53 by curcumin in induction of HSC senescence. In this study, our evidence showed that curcumin induced the expression of P53 through activation of PPAR*γ* in activated HSCs. The previous studies have shown that the expression of PPAR*γ* was diminished when quiescent HSCs become activated. At the same time, activation of PPAR*γ* was required for curcumin to reduce cell proliferation, induce apoptosis and suppress ECM gene expression.^15^ In the present study, we found that curcumin activated PPAR*γ* and promoted the nuclear translocation of PPAR*γ*. Moreover, binding to DNA sequence and transactivated P53 by PPAR*γ* were all significantly enhanced in curcumin-treated HSCs. Our further experiments revealed that PPAR*γ* played a key role in curcumin induction of HSC senescence. PPAR*γ* agonist 15d-PGJ2 enhanced the curcumin induction of expression of senescence markers. Besides, PPAR*γ* antagonist PD68235 abrogated this stimulatory effect. We also examined the effect of PPAR*γ* on the expression of P53 in senescent HSCs. Real-time PCR, western blot and immunofluorescence staining consistently showed that curcumin could promote the expression of P53 by activating PPAR*γ*. Taken together, these data revealed that activation of PPAR*γ* causally stimulated P53 expression, which critically led to the cell growth arrest and senescence of activated HSCs.

In summary, the aggregate data in this study demonstrated that curcumin regulated senescence by inducting PPAR*γ*/P53 pathway in activated HSCs (Figure 6i), indicating that induction of cellular senescence could be a new strategy for inhibiting fibrogenic properties in activated HSCs by curcumin. These findings provided novel molecular basis for the development of curcumin as a promising antifibrotic agent for liver fibrosis.

## Materials and Methods

### Reagents and antibodies

The following compounds were used in this study: curcumin, compounds 15d-PGJ2 and PD68235 (Sigma, St. Louis, MO, USA); Etoposide (Selleckchem, Houston, TX, USA); P53 inhibitor PFT-*α* (Nanjing EnoGene Biotech Co., Ltd, Nanjing, China). All these compounds were dissolved in dimethylsulfoxide (DMSO; Sinopharm Chemical Reagent Co., Ltd, Shanghai, China) for experiments. The primary antibodies were used in this study: P16, P53, cyclin D1, cyclin E1, CDK4, CDK6, PPAR-*γ*, *γ*H2AX and *β*-actin (Cell Signaling Technology, Danvers, MA, USA); P21 (Santa Cruz Biotechnology, Santa Cruz, CA, USA); Hmga1 (Abcam Technology, Abcam, Cambridge, UK); *α*1(I)-procollagen and *α*-SMA (Epitomics, San Francisco, CA, USA).

### Experimental animal procedures

All experimental procedures were approved by the institutional and local committee on the care and use of animals of Nanjing University of Chinese Medicine (Nanjing, China), and all animals received humane care according to the National Institutes of Health (USA) guidelines. Male Sprague Dawley rats (200–250 g body weight) were obtained from Shanghai Slac Laboratory Animal (Shanghai, China). A mixture of carbon tetrachloride (CCl_4_; 0.1 ml per 100 g body weight) and olive oil (1 : 1 (v/v)) was used to induce liver fibrosis in rats. A total of 36 rats were randomly divided into six groups (*n*=6). Group 1 was the vehicle control in which rats were not administrated CCl_4_ or curcumin but intraperitoneally (i.p.) injected with olive oil. Group 2 was the CCl_4_ group in which rats were i.p. injected with CCl_4_ without curcumin treatment. Group 3 was the positive control in which rats were injected with CCl_4_ and treated with colchicine (Yifeng Pharmacy, Nanjing, China) at 0.1 mg/kg. Groups 4, 5 and 6 were treatment groups in which rats were i.p. injected with CCl_4_ and orally given curcumin at 100, 200 and 300 mg/kg, respectively. Rats in groups 2–6 were i.p. injected with CCl_4_ every other day for 8 weeks. Curcumin and colchicine were suspended in sterile phosphate-buffered saline (PBS) and given once daily by gavage during weeks 5–8. The control animals in groups 1 and 2 were similarly handled, including i.p. injection with the same volume of olive oil and oral administration of the same volume of PBS. At the end of experiments, rats were killed after being anesthetized by i.p. injection with pentobarbital (50 mg/kg). A small portion of the liver was removed for histopathological and immunohistochemical studies.

Male ICR mice were randomly divided into five groups (*n*=10) and given administration of Ad.Fc (a control adenovirus encoding IgG2*α* Fc fragment), curcumin (300 mg/kg, once per two days) plus Ad.Fc, curcumin plus Ad.shP53 (adenovirus encoding mouse P53 shRNA for inhibiting P53 pathway), or Ad.shP53, respectively, throughout the 8-week period of CCl_4_ treatment and the control group with no treatment. Adenoviruses (2.5 × 10^7^ pfu/g, once per 2 weeks) were injected into mice by tail vein. A mixture of carbon tetrachloride (CCl_4_; 0.5 ml per 100 g body weight) and olive oil (1 : 9 (v/v)) was used to induce liver fibrosis in mice by i.p. injection. After 8 weeks, liver were fixed in 4% buffered paraformaldehyde for histological analysis of liver fibrosis and immunostaining analysis or HSCs were isolated for western blot analysis.

### Cell culture

HSC-T6 cell line was purchased from Cell Bank of Chinese Academy of Sciences (Shanghai, Chinese). Cells were cultured in Dulbecco's modified Eagle's medium (DMEM; Invitrogen, Grand Island, NY, USA) with 10% fetal bovine serum (FBS; Sijiqing Biological Engineering Materials, HangZhou, China), 1% antibiotics and grown in a 95% air and 5% CO_2_ humidified atmosphere at 37 °C.

### Immunofluorescence staining

Immunofluorescence staining with liver tissues or treated cells were performed as we previously reported.^20^ 4′,6-Diamidino-2-phenylindole (DAPI) was used to stain the nucleus in liver tissues. Hoechst reagent was used to stain the nucleus of HSCs *in vitro*. All the images were captured with the fluorescence microscope and representative images were shown. The software Image J was used to quantitate the fluorescent intensity on the micrographs.

### Cell proliferation assay and apoptosis assay

HSCs were treated with various reagents at indicated concentrations for 24 h. Cell proliferation was evaluated using Cell Counting Kit-8 (Beyotime Biotechnology, Haimen, China) according to the protocols. Morphology of apoptotic HSCs was evaluated using TUNEL staining kits and DAPI staining kits (Nanjing Keygen Biotechnology Co., Ltd, Nanjing, China) according to the protocols. All these experiments were performed in triplicate.

### Analysis of HSC senescence

HSC senescence was determined by the detection of SA-*β*-gal (senescence-associated *β*-galactosidase) activity using an SA-*β*-gal staining kit (Cell Signaling). Briefly, adherent cells were fixed with 0.5% glutaraldehyde in PBS for 15 min, washed with PBS containing 1 mM MgCl_2_ and stained overnight in PBS containing 1 mM MgCl_2_, 1 mg/ml X-Gal, 5 mM potassium ferricyanide and 5 mM potassium ferrocyanide. All the images were captured with a light microscope and representative images were shown. Results were from triplicate experiments.

### Cell transfection with P53 siRNA

P53 siRNA (Santa Cruz Biotechnology; sc-45917) of 5 *μ*g was added to 150 *μ*l medium without serum and antibiotic and incubated at room temperature for 5 min. LipofectAMINE reagent (Life Technologies, Grand Island, NY, USA) of 3 *μ*l was added to 150 *μ*l medium without serum and antibiotics and incubated at room temperature for 5 min. The above two solutions were mixed well at room temperature for 20 min and about 300 *μ*l transfection complex was obtained. Cells were incubated with the transfection complex solution at 37 °C for 8 h, and then were re-incubated in complete medium at 37 °C for an additional 16 h. Control siRNA (Santa Cruz Biotechnology; sc-37007) is a non-targeting 20–25 nt siRNA designed as a negative control.

### Cell cycle analysis by flow cytometry

Distribution of cell cycle was determined by PI staining and flow cytometry analysis. HSCs were seeded in six-well plates and cultured in DMEM supplemented with 10% FBS for 24 h, and then were treated with DMSO, etoposide and curcumin at indicated concentrations for 24 h. Cells were then harvested and fixed, and the cell cycle was then detected by the cellular DNA flow cytometric analysis kit (Nanjing Keygen Biotech Co., Ltd) according to the protocol. Percentages of cells within cell cycle compartments (G0/G1, S and G2/M) were determined by flow cytometry (FACS Calibur; Becton, Dickinson and Company, Franklin Lakes, NJ, USA). The data were analyzed using the software Cell Quest. Results were from triplicate experiments.

### Liver histopathology

Harvested liver tissues were fixed in 10% neutral-buffered formalin and embedded in paraffin. Liver slices of 50 *μ*m thick were prepared and stained with H&E and Masson's trichrome stain by using standard methods. For sirius red collagen staining, thin sections were deparaffinized and stained with picro-sirius red for 1 h at room temperature. After washes, sections on the slides were dehydrated in 100% ethanol and in xylene, and then they were mounted in Permount. IHC staining was performed to detect the expression of P16 using an IHC accessory kit (Bethyl Laboratories, Montgomery, TX, USA). Photographs were taken in a blinded fashion at random fields. Representative views of liver sections are shown.

### Real-time PCR

Total RNA was prepared from treated HSCs using Trizol reagent (Sigma) following the protocol provided by the manufacturer. Real-time PCR was performed as we described previously.^35^ Glyceraldehyde phosphate dehydrogenase (GAPDH) was used as the invariant control. Fold changes in the mRNA levels of target genes related to the invariant control GAPDH were calculated according to the suggested method.^36^ The following primers of genes (GenScript, Nanjing, China) were used: *α*-SMA: (forward) 5′-CCGACCGAATGCAGAAGG-3′, (reverse) 5′-ACAGAGTATTTGCGCTCCGGA-3′ *α*1(I)-procollagen: (forward) 5′-CCTCAAGGGCTCCAACGAG-3′, (reverse) 5′-TCAATCACTGTCTTGCCCCA-3′ P16: (forward) 5′-CCGAGAGGAAGGCGAACTC-3′, (reverse) 5′-GCTGCCCTGGCTAGTCTATCTG-3′ P21: (forward) 5′-GAGCAAAGTATGCCGTCGTC-3′, (reverse) 5′-CTCAGTGGCGAAGTCAAAGTTC-3′ HMGA1: (forward) 5′-CAGGAAAAGGATGGGACTGA-3′, (reverse) 5′-CTTGTTCTTGCTTCCCTTCG-3′ P53: (forward) 5′-GCCATCTACAAGAAGTCACAGC-3′, (reverse) 5′-GATGATGGTAAGGATAGGTCGG-3′ GAPDH: (forward) 5′-GACATCAAGAAGGTGGTGAAGC-3, (reverse) 5′-TGTCATTGAGAGCAATGCCAGC-3. Results were from triplicate experiments.

### Western blot analyses

Whole-cell protein extracts were prepared from passaged HSCs. The protein levels were determined using a BCA assay kit (Pierce, Rockford, IL, USA). Proteins (50 *μ*g/well) were separated by SDS-polyacrylamide gel, transferred to a PVDF membrane (Millipore, Burlington, MA, USA), blocked with 5% skim milk in Tris-buffered saline containing 0.1% Tween 20. Target proteins were detected by corresponding primary antibodies, and subsequently by horseradish peroxidase-conjugated secondary antibodies. Protein bands were visualized using chemiluminescence reagent (Millipore). Equivalent loading was confirmed using an antibody against *β*-actin. The levels of target protein bands were densitometrically determined using Quantity One 4.4.1. The variation in the density of bands was expressed as fold changes compared with the control in the blot after normalized to *β*-actin. Representative blots were from three independent experiments.

### Enzyme-linked immunosorbent assay (ELISA)

The levels of PPAR*γ* in HSCs were determined using an ELISA kit (Nanjing Jiancheng Bioengineering Institute, Nanjing, China) according to the protocol. Six duplicate wells were set up for each group. Results were from triplicate experiments.

### Electrophoretic mobility shift assay (EMSA)

Nuclear extracts from treated HSCs were prepared using the NE-PER Nuclear Protein Extraction Kit (Pierce) according to the protocol. The biotin-labeled PPAR*γ* probe was prepared using an EMSA kit in accordance with the manufacturer's instructions (Pierce). The extracted nuclear protein (10 mg) was incubated in a binding reaction mixture containing 1.5 ml 10 × binding buffer, 1.5 ml poly (dI−dC) (1.0 mg/ml) and ddH_2_O to a final volume of 14.4 ml for 20 min at room temperature. Then, the probe of 0.6 ml (300 fmol) was added and incubated for 20 min at room temperature. Where indicated, 2 ml of specific, cold-competitor oligonucleotides in 100 × competing buffer was added before the labeled probe, and the reaction was incubated for 20 min. Protein–DNA complexes were subjected to electrophoresis in a 6.5% acrylamide gel at 4 °C for 1 h. The gels were transferred to the bonding membrane at room temperature for 40 min. After crosslinking for 10 min with an ultraviolet crosslinking apparatus, the membrane was blocked, streptavidin-HRP labeled, washed again and equilibrated. Images were captured using the Gel Doc2000 system (Bio-Rad, Hercules, CA, USA).

### Dual-luciferase reporter assay

The luciferase reporter plasmid P53 (pP53-TA-luc) was purchased from Beyotime Biotechnology. Transfection efficiency was determined by co-transfection of a Renilla luciferase reporter (pRL-TK; Promega, Madison, WI, USA), pRL-TK Vector (0.5 *μ*g/well). Luciferase activities were measured using a Dual-Luciferase Reporter Assay System (Promega), according to the manufacturer's instructions. Luciferase assays were performed as described previously.^37^ Each treatment was performed in triplicate. Results from three independent experiments were combined.

### Statistical analysis

Data were presented as mean±s.d., and results were analyzed using SPSS16.0 software. The significance of difference was determined by one-way ANOVA with the *post-hoc* Dunnett's test. Values of *P*<0.05 were considered to be statistically significant.

## Abbreviations

*α*-SMA: *α*-smooth muscle actin
CDKs: cyclin-dependent kinases
DMEM: Dulbecco's modified Eagle's medium
DMSO: dimethylsulfoxide
ECM: extracellular matrix
FBS: fetal bovine serum
GAPDH: glyceraldehyde phosphate dehydrogenase
HSCs: hepatic stellate cells
PBS: phosphate-buffered saline
PFT-*α*: pifithrin-*α*
PPAR*γ*: peroxisome proliferator activated receptor *γ*
SA-*β*-Gal: senescence-associated *β*-galactosidase
